# F135. BODY MASS INDEX TRAJECTORIES IN CHILDHOOD AND RISK FOR NON-AFFECTIVE PSYCHOSIS – A GENERAL POPULATION COHORT STUDY

**DOI:** 10.1093/schbul/sby017.666

**Published:** 2018-04-01

**Authors:** Elina Sormunen, Maiju Saarinen, Raimo K R Salokangas, Jorma Viikari, Olli Raitakari, Jarmo Hietala

**Affiliations:** 1 University of Turku; 2 University of Turku, Turku University Hospital

## Abstract

**Background:**

It is well known that underweight in adolescence and early adulthood predicts later schizophrenia.1 Some studies have shown an association between future schizophrenia or psychosis and underweight in children, starting at the age of 7.2 There are very few previous studies concerning underweight in early childhood and the risk of psychosis as well as other psychiatric outcomes. Our aim was to study whether deviation from normal weight, i.e. underweight or overweight, in early childhood and adolescence predicts later development of non-affective psychosis. And if so, whether the mechanism is specific to psychosis or also predicts other psychiatric disorders.

**Methods:**

The participants were derived from a general population cohort study ‘Cardiovascular Risk of Young Finns’, which was started in 1980 with 3596 children and adolescents participating from six different age groups (3–18 years), with a continued follow-up. BMI was recorded before the first hospitalization due to a psychiatric disorder (≤18 years of age) and categorized as underweight, normal weight or overweight using the BMI classification for children and adolescents provided by Cole et al.3,4 All psychiatric diagnoses of the participants were acquired from the Finnish Hospital Discharge Register. We formed DSM-IV diagnostic groups of non-affective psychosis (n=70, including a schizophrenia subgroup, n=41), personality disorders (n=44), affective disorders (mood- and anxiety disorders, n=115), and substance-related disorders (n=53). Participants in the diagnostic groups were compared with subjects with no psychiatric diagnoses (n=3313). Sex, age, low birth weight and mother’s mental disorders were used as potential confounders in the analyses.

**Results:**

Underweight, but not overweight, during the age of 3 to 18 years independently predicted later development of non-affective psychosis. Underweight in childhood and/or adolescence increased the risk of psychosis over two-fold (relative risk (RR) [95% CI] 2.31 [1.2–4.4]). Results were similar for schizophrenia; underweight was associated with nearly 2.5-fold risk of schizophrenia (RR 2.44 [1.03–5.8]). Underweight or overweight in childhood and adolescence was not significantly associated with any other studied psychiatric disorder with a more severe clinical phenotype that required hospital treatment.

**Discussion:**

Underweight in childhood and adolescence is an independent risk factor for later non-affective psychosis. The mechanism behind underweight in premorbid phase of psychosis is not known but e.g. low level of insulin-like growth factor-I (IGF-I) may be involved. These results support the hypothesis of schizophrenia as a neurodevelopmental disorder with somatic aspects appearing already in early childhood and psychosis as a late stage of illness.

**References:**

1.Weiser M, Knobler H, Lubin G, et al. Body mass index and future schizophrenia in Israeli male adolescents. J Clin Psychiatry. 2004;65(11):1546–1549. doi:10.4088/JCP.v65n1117.

2.Wahlbeck K, Forsén T, Osmond C, Barker DJP, Eriksson JG. Association of Schizophrenia With Low Maternal Body Mass Index, Small Size at Birth, and Thinness During Childhood. Arch Gen Psychiatry. 2001;58(1):48. doi:10.1001/archpsyc.58.1.48.

3.Cole TJ. Establishing a standard definition for child overweight and obesity worldwide: international survey. Bmj. 2000;320(7244):1240-1240. doi:10.1136/bmj.320.7244.1240.

4.Cole TJ, Flegal KM, Nicholls D, Jackson AA. Body mass index cut offs to define thinness in children and adolescents: international survey. Bmj. 2007;335(7612):194-194. doi:10.1136/bmj.39238.399444.55.

